# Proteomic Deep Mining the Venom of the Red-Headed Krait, *Bungarus flaviceps*

**DOI:** 10.3390/toxins10090373

**Published:** 2018-09-13

**Authors:** Alex Chapeaurouge, Andreza Silva, Paulo Carvalho, Ryan J. R. McCleary, Cassandra Marie Modahl, Jonas Perales, R. Manjunatha Kini, Stephen P. Mackessy

**Affiliations:** 1 Fundação Oswaldo Cruz-Ceará, Rua São José, 2º Pavimento, Precabura, Eusébio 61760-000, Brazil; 2 Laboratório de Toxinologia, Instituto Oswaldo Cruz, Fiocruz, Rio de Janeiro 21045-900, Brazil; ann-x3@hotmail.com (A.S.); jperales@ioc.fiocruz.br (J.P.); 3 Computational Mass Spectrometry& Proteomics Group, Carlos Chagas Institute, Fiocruz, Paraná 81350-010, Brazil; paulo@pcarvalho.com; 4 Department of Biology, Stetson University, 421 N. Woodland Blvd, DeLand, FL 32723, USA; rmccleary@stetson.edu; 5 Department of Biological Sciences, National University of Singapore, 14 Science Drive 4, Singapore 117543, Singapore; dbscmm@nus.edu.sg (C.M.M.); dbskinim@nus.edu.sg (R.M.K.); 6 School of Biological Sciences, University of Northern Colorado, 501 20th St., CB 92, Greeley, CO 80639-0017, USA

**Keywords:** *Bungarus flaviceps*, enzymes, Red-headed Krait, snake venom, toxins, proteome

## Abstract

The use of -omics technologies allows for the characterization of snake venom composition at a fast rate and at high levels of detail. In the present study, we investigated the protein content of Red-headed Krait (*Bungarus flaviceps*) venom. This analysis revealed a high diversity of snake venom protein families, as evidenced by high-throughput mass spectrometric analysis. We found all six venom protein families previously reported in a transcriptome study of the venom gland of *B. flaviceps*, including phospholipases A_2_ (PLA_2_s), Kunitz-type serine proteinase inhibitors (KSPIs), three-finger toxins (3FTxs), cysteine-rich secretory proteins (CRISPs), snaclecs, and natriuretic peptides. A combined approach of automated database searches and de novo sequencing of tandem mass spectra, followed by sequence similarity searches, revealed the presence of 12 additional toxin families. De novo sequencing alone was able to identify 58 additional peptides, and this approach contributed significantly to the comprehensive description of the venom. Abundant protein families comprise 3FTxs (22.3%), KSPIs (19%), acetylcholinesterases (12.6%), PLA_2_s (11.9%), venom endothelial growth factors (VEGFs, 8.4%), nucleotidases (4.3%), and C-type lectin-like proteins (snaclecs, 3.3%); an additional 11 toxin families are present at significantly lower concentrations, including complement depleting factors, a family not previously detected in *Bungarus* venoms. The utility of a multifaceted approach toward unraveling the proteome of snake venoms, employed here, allowed detection of even minor venom components. This more in-depth knowledge of the composition of *B. flaviceps* venom facilitates a better understanding of snake venom molecular evolution, in turn contributing to more effective treatment of krait bites.

## 1. Introduction

In the last decade, there has been a tremendous increase in the knowledge of snake venom composition and evolution, mainly because of the application of “omics” techniques, in particular, high-throughput transcriptomic investigations of venom gland tissue in combination with proteomic studies of venom [1,2,3,4,5,6]. The use of these highly sensitive technologies now makes it feasible to study the venom gland transcriptomes/proteomes of virtually any snake species in detail, even those producing low venom yields, such as kraits and many rear-fanged colubrid snakes. Recently, Viala et al. investigated the venom of the Australian Eastern Brown Snake (*Pseudonaja textilis*) by combining high throughput proteomics and transcriptomics techniques [7]. Their deep data-mining approach led to the identification of a new transcript variant of venom coagulation factor 5a. The authors also concluded that splicing variants represent an important source of toxin diversification. Likewise, a comparative deep venomics study including venom gland transcriptomes and proteomes of the Australian western brown snakes *Pseudonaja aspidorhyncha*, *Pseudonaja nuchalis*, and *Pseudonaja textilis* revealed not only accelerated evolution of different toxin families, but also a wide array of novel peptide sequences from six bioactive peptide families [8]. In addition, an integrated analysis of the transcriptome and proteome of the Amazon Puffing Snake *Spilotes sulphureus* identified two novel three-finger toxins: sulditoxin and sulmotoxin-1 [9]. These two toxins not only represent the most abundant venom proteins, but also showed distinct prey-specific toxicities. While sulditoxin exhibits high toxicity towards lizard prey, but is non-toxic towards mammalian prey, sulmotoxin 1 shows the reverse trend [9]. The authors reasoned that this is the first example in which a contrasting pattern of toxicity in relation to diet has been shown for proteins in the venom of a single snake. As these technologies are applied to poorly known venoms, new toxins may be discovered, as demonstrated by an investigation of the proteome and transcriptome of the venom of the Australian elapid snake *Drysdalia coronoides*, which revealed, among other protein families, novel three-finger toxins (3FTx) proteins [10]. These unique sequences could indicate novel biological functions related to specific types of prey consumed. For example, Brown Treesnake (*Boiga irregularis*), and Green Vinesnake (*Oxybelis fulgidus*), two rear-fanged colubrid snakes, both feed primarily on lizards and birds, and their venoms contain different novel 3FTxs that are specific for these taxa and are harmless toward mammals [11,12], strongly indicating selection for taxon-specific venom toxins. These examples point to the significance of deep mining venomics studies by, for example, combined transcriptome and proteome investigations to detect even less abundant venom sequences that might be of biological or pharmaceutical importance. In the present study, we investigated the venom proteome of *B. flaviceps* by a complementary approach of electrospray and MALDI mass spectrometry (MS) to probe deeply for even minor venom compounds.

The *Bungarus* genus (kraits) belongs to the family Elapidae. To date, 15 different species of this genus have been recognized. The genus *Bungarus* was notorious during the Vietnam War, and American troops referred to these snakes as the “two-step snake” in the mistaken belief that after being bitten, a person was only two steps away from death [13]. Investigations of venom gland transcriptomes [14] and proteomes [15] of *B. multicinctus* revealed that tandem duplications contributed significantly to the expansion of toxin multigene families in the transcriptome. Interestingly, the venom proteome [15] appeared to be less diverse than the corresponding transcriptome of *B. multicinctus* [14]. In addition, recent proteomic studies of the Malaysian kraits *B. fasciatus* and *B. candidus* led to the identification of natriuretic peptides, vespryns, and serine proteases, protein families previously not detected in these species. The venoms of the Vietnamese kraits *B. multicinctus* and *B. fasciatus* showed a pronounced difference in the amounts of three-finger toxins, which were highly abundant in the former venom (28%), but, surprisingly, were detected in very low amounts in the latter (1%). A recent study of venom variation in *Bungarus caeruleus* (Common Krait) concluded that Pakistani *B. caeruleus* should be incorporated into the Indian Vins Polyvalent Antivenom (VPAV), because krait bites at this locality showed only moderate neutralization by VPAV [16].

**Figure 1:**
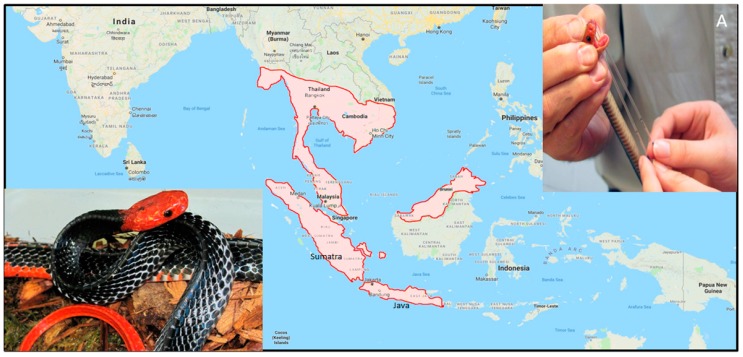
Approximate distribution of the Red-headed Krait (*Bungarus flaviceps*). Inset **A** shows the extraction of venom from *B. flaviceps*, a low-yielding front-fanged snake. Distributions are adapted from the literature ([17], 2010, New Holland Publishers) and the map is from Google Earth.

*Bungarus flaviceps*, the Red-headed Krait, is widely distributed across southeast Asia, from southern Myanmar and Thailand south to Borneo and Java [17] (Figure 1).

Initially, the transcriptome of *B. flaviceps* was investigated by sequencing 845 expressed sequence tags (ESTs) from a single venom gland of a *B. flaviceps* specimen. Roughly 75% of the transcripts were putative toxins and six different protein venom families were identified. However, the EST method has rather low resolution, especially when compared with next-generation high-throughput DNA sequencing techniques [8], which provide a deeper understanding of the venom composition.

*Bungarus flaviceps* feeds primarily on other snakes and on lizards [17], so its venom may also show novel toxins or specific compositional/structural motifs targeting these preferred prey. As *B. flaviceps* does not feed on mammals, but only on ectothermic reptiles, we might anticipate that specialized toxins may have evolved that more effectively incapacitate ectothermic prey. Venoms of several species of the genus *Bungarus* have been studied, and a general characteristic is a high content of potent neurotoxins, among which α-, β-, and κ-bungarotoxins are the most well-known [18,19,20]. The mass spectrometric data of the present study were analyzed by automated searches against a custom snake venom database, as well as by de novo sequencing of the tandem mass spectra followed by sequence-similarity analysis. These investigations revealed the presence of 18 snake venom protein families as evidenced by the detection of corresponding peptides, including protein families not previously detected in *Bungarus* venom. The results of this study have direct implications for envenomation treatment and for molecular venom evolution, particularly among elapid species.

## 2. Results and Discussion

The venom proteome of the elapid *B. flaviceps* was investigated by mass spectrometric analysis using complementary ionization techniques, including electrospray and MALDI. Based on the results of database searches and BLAST analyses of de novo sequenced tandem mass spectra, we were able to provide a comprehensive overview of the different protein families present in the venom. In addition, spectral counts of the tandem MS (MS/MS) data matched to known toxin sequences yielded a quantitative estimate of the different protein families in the venom. In total, 88 different proteins were identified that belong to 18 distinctive protein families; a similar number of proteins was found for the venoms of *B. candidus* and *B. fasciatus* [21]. Below, we discuss the different toxin families of *B. flaviceps* venom in detail.

### 2.1. Major Venom Components

**Figure 2:**
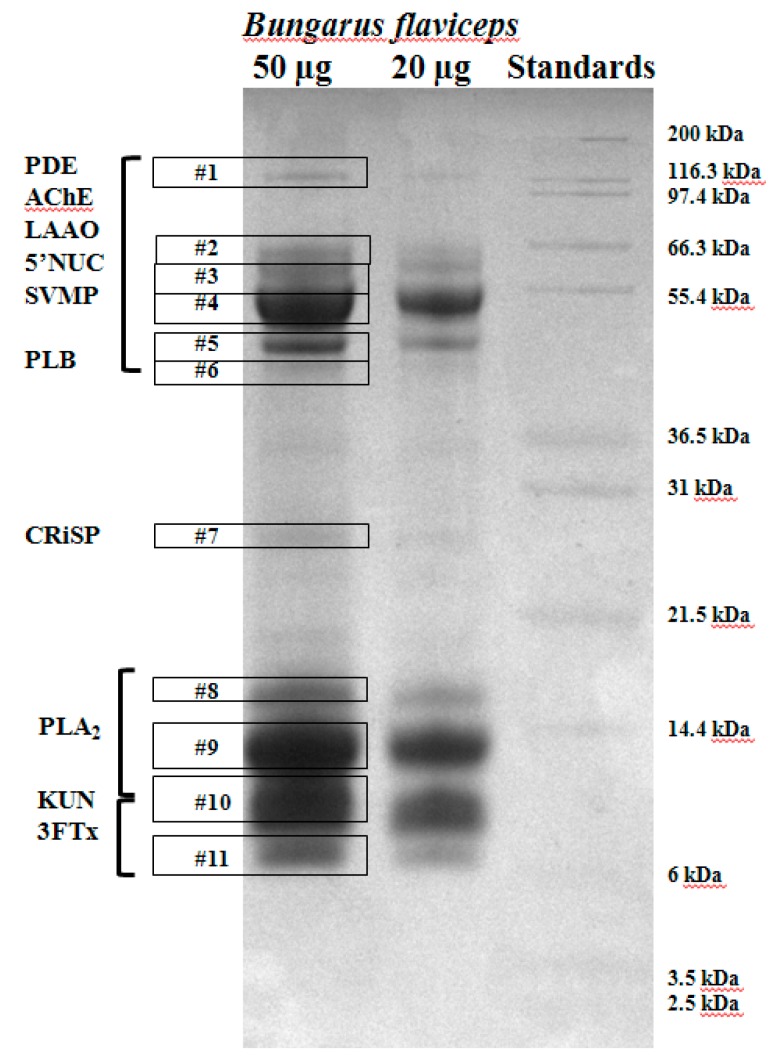
One-dimensional gel of the crude venom of *B. flaviceps*. Prominent venom protein families (boxed) are based on their molecular masses and confirmed by liquid chromatography (LC)-tandem mass spectrometry (MS/MS) (see also Table S1). Bands were excised for further analysis. PDE—phosphodiesterases; AChE—acetylcholinesterases; LAAO—L-amino-acid oxidase; 5’-NUC—5’inucleotidase; SVMP—snake venom metalloproteinase; PLB—phospholipase B; CRISP—cysteine-rich seceretory proteins; PLA_2_—phospholipase A; KUN—Kunitz-type serine proteinase inhibitor; 3FTx—three-finger toxin.

**Figure 3:**
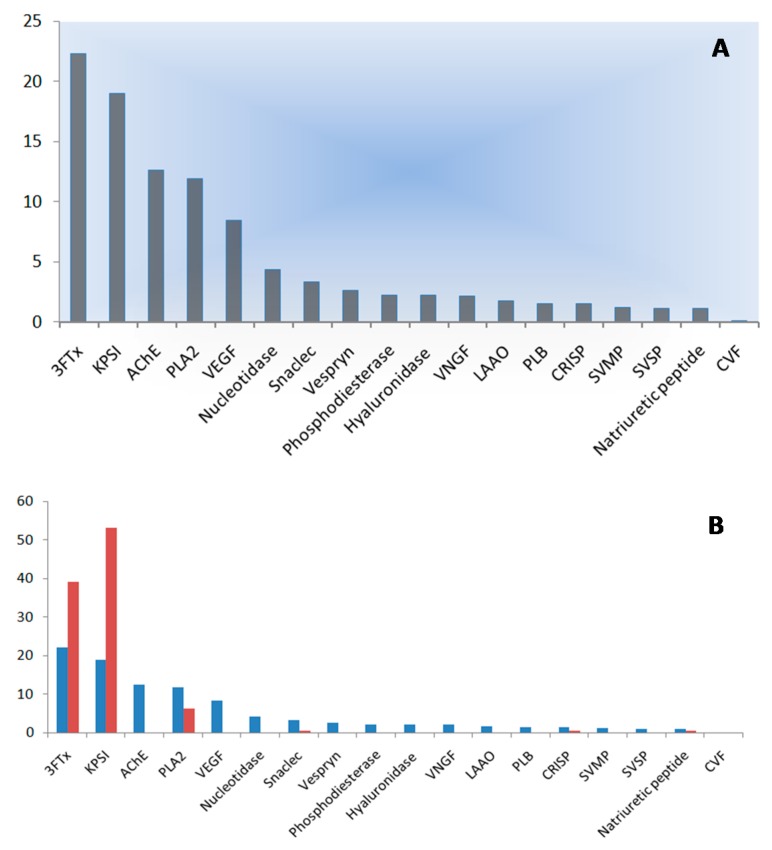
(**A**) Abundances of the venom protein families of *B. flaviceps* as evidenced by normalized mass spectrometric spectral count. (**B**) Comparison of abundances of venom protein families (*B. flaviceps*) by transcriptomic (red, adapted from [25], 2010, Springer Nature) and proteomic analysis. KSPI—Kunitz-type serine proteinase inhibitors; VEGF—vascular endothelial growth factor; VNGF—venom nerve growth factor; SVSP—snake venom serine proteinase; CVF—cobra venom factor.

The most abundant components of the venom of *B. flaviceps* (22.3%; Figure 2 and Figure 3) are represented by 3FTxs, similar to that observed for the related *B. fasciatus*, in which 3FTxs make up 30% of the venom content [21]. As a non-enzymatic snake venom protein family, 3FTxs are structurally characterized by three-stranded loops protruding from a central core of the molecule. In spite of their similarity in structure and relatively small size (<10 kDa), 3FTxs have remarkable differences in function, including platelet aggregation, neurotoxicity, and cytotoxicity [22,23,24]. Furthermore, 3FTxs were identified as the major component of the *B. flaviceps* venom at the transcript level [25] and included both non-conventional and short-chain isoforms. We confirmed the presence of most of these 3FTxs in the proteome (Table 1), in addition to a protein similar to the κ-bungarotoxin from *B. multicinctus* (Table 1). However, no cytotoxins or cardiotoxins were encountered in the venom of *B. flaviceps*, consistent with the results of a recent analysis of *B. caeruleus* venom, in which these proteins were also not detected [16]. Interestingly, we were not able to demonstrate the presence of α-bungarotoxin, a 3FTx detected in the venoms of the congeneric species *B. candidus*, *B. fasciatus* [21], and *B. multicinctus* [26]. As it was also not detected in the transcriptome study of *B. flaviceps* [25], we speculate that its absence could indicate a differential need of suite of toxins for prey taken by *B. flaviceps*. Alternatively, it might indicate a random loss of the gene during the differentiation of *B. flaviceps* from a last common ancestor.

Serine proteinase inhibitors were the second most abundant toxin family in the venom of *B. flaviceps* and accounted for 19% of the total spectral count (Figure 3). Kunitz-type serine proteinase inhibitors (KSPI) carry a conserved Kunitz motif similar to that found in bovine pancreatic trypsin inhibitors [27,28]. They not only inhibit trypsin and chymotrypsin [29,30], but also cause different pharmacological effects, including anticoagulation and fibrinolysis. At the transcript level, six isoforms of KPSI were detected [25], while the proteome analysis revealed the presence of three different isoforms, suggesting that three isoforms are not expressed in the venom. However, the difference in the number of isoforms detected in the complementary studies might be related to poor ionization of unique peptides of the corresponding isoform rather than the absence of this protein in the venom. The B-chain of β-bungarotoxin exhibits high homology with KSPIs and, therefore, has been grouped together with this toxin family. At the protein level, we found four different isoforms of the B-chain (Table 1 and Table 2), but only one of those matched one of the four isoforms discovered in the transcriptome [25], perhaps because of differences in snakes sampled in the two studies or because of the lack of fine scale transcriptomic resolution used by Siang et al.

Acetylcholinesterases (AChEs) are important regulators of neurotransmission at the neuromuscular junction, and they rapidly hydrolyze acetylcholine. Acetylcholinesterases were the third most abundant toxin family in the venom (12.6%; Figure 3). The function of this protein family in snake venoms is still poorly understood, but it may potentiate the action of α-neurotoxins at the nAChR of the motor endplate. BfAChE was identified with relatively high sequence coverage, consistent with the view that only a single isoform of this protein family is present in the venom of *B. flaviceps* [25]. Of note is that AChEs in related Bungarus venoms are less abundant. For example, Malaysian *B. candidus* and *B. fasciatus* venoms revealed relative abundances of 4.9% and 4.7%, respectively [21]. In addition, AChEs were detected in very low abundances (1.1%) in the Vietnamese *B. multicinctus* venom [26]. This might indicate that AChEs play a more important role in envenomation in *B. flaviceps* than in congeneric venoms.

Phospholipases A_2_ (PLA_2_s) are broadly occurring esterolytic enzymes, and in venoms, they cause diverse pharmacological consequences including myotoxic, cardiotoxic, and anticoagulant pathologies, as well as pre- and postsynaptic neurotoxic effects [31]. While PLA_2_s represent the fourth most abundant component of the venom of *B. flaviceps* (11.9%; Figure 3), this protein family is considerably more abundant in *B. fasciatus* and *B. candidus* venoms, where PLA_2_s make up 44% and 25%, respectively, of the venom content [21]. The A chain of β-bungarotoxin, a heterodimeric protein with presynaptic neurotoxic effects [20], shows pronounced sequence similarity to PLA_2_s and we were able to detect two isoforms of the A chain in the venom (Table 1), while in the transcriptome, three isoforms were detected. Given that β-bungarotoxins are the main lethal components in *B. multicinctus* and *B. candidus*, the venom of *B. flaviceps* is most likely potently neurotoxic, with envenomation effects similar to those of other kraits. Additionally, we not only confirmed the presence of PLA_2_s also observed at the transcript level [25], but also found peptides homologous to those of PLA_2_s isolated from divergent elapid species, including *B. fasciatus*, *B. candidus*, *Austrelaps superbus*, *Micropechis ikaheka*, *Laticauda colubrina*, and *L. semifasciata*. This indicates that PLA_2_s are more diversified in *B*. *flaviceps* venom than was initially appreciated, and they are likely functionally important venom components. 

Vascular endothelial growth factor (VEGF) is an important angiogenic factor in mammals and is produced by various cell types, including macrophages [32], platelets [33], and tumor cells [34]. VEGFs have also been identified in snake venoms (known as VEGF-F) and they have been found to strongly stimulate proliferation of vascular endothelial cells in vitro, as well as to induce hypotension in rats [35]. Four peptides that matched the sequence of a hypothetical venom protein of *Ophiophagus hannah* (King Cobra) were identified in the present study. As this hypothetical protein also shares about 80% sequence homology with a VEGF-F from the viper *Vipera ammodytes* [36], it is highly probable that VEGF-Fs are also part of the venom proteome of *B. flaviceps*.

Nucleotidases in snake venoms are believed to catalyze the production of adenosine, which could lead to paralysis and prey immobilization [37], but their role in envenomation is still unclear. Tryptic peptides derived from *B. flaviceps* venom matched two isoforms of nucleotidases originally isolated from venoms of the elapid *Micrurus fulvius* [38] and the colubrid *Boiga irregularis* [39]. Nucleotidases are typically found in low abundance in snake venoms, and it would be of interest to investigate their biological functions.

Snaclecs are mostly heterodimeric proteins that contain the same three-dimensional fold as classical C-type lectins [40]. Through binding to the von Willebrand factor or to different collagen receptors, they affect platelet activation. In this study, we detected peptides both identical to and similar to snaclec transcripts from *B. fasciatus*, *Micrurus fulvius*, and *Bothrops jararaca*. This appears to be the first identification of this protein family at the protein level in the genus *Bungarus*. Indeed, several reports found evidence of snaclecs in the venom glands of *B. flaviceps* [25], as well as *B. fasciatus* and *B. multicinctus* [41] at the transcript level, but to our knowledge, this is the first demonstration of snaclecs in the *Bungarus* proteome. 

The venom of *O. hannah* was the original source of a novel snake venom family known as the vespryns [42]. Functionally, these proteins have been described to induce hypolocomotion and hyperalgesia in mice [42]. Vespryns matching those from several species, including *O. hannah*, *Pseudechis australis*, and *Drysdalia coronoides*, were encountered in the present study. Recently, vespryns were also found in the venom of *B. candidus* [21], but tryptic peptides of *B. flaviceps* origin did not match these proteins.

Phosphodiesterases (PDE) are basic enzymes with molecular masses in the range of 98 to 140 kDa [43], and they catalyze the hydrolysis of phosphodiester bonds from the 3’ terminus of polynucleotides. Though PDEs have long been known to be present in many venoms, the first complete primary structure of a PDE from snake venom was only recently published [44]. We encountered sequences similar to PDEs of the elapid *Micrurus fulvius* and of several different pitvipers (Table 1 and Table 2). These results further corroborate the presence of PDE in *Bungarus* venoms, as they were also reported in the venom of *B. fasciatus* [21].

Hyaluronidases from snake venoms are endo-β-*N*-acetyl-hexosaminidases with molecular masses in the range of 30 to 110 kDa [43]. These enzymes cleave hyaluronan, a major glycosaminoglycan constituent of the extracellular matrix, into *N*-acetylglucosamine and oligocarbohydrates. Therefore, hyaluronidases have been implicated as important factors for the distribution and dissemination of the venom in tissues and are often called “spreading factors” [43]. Peptides with pronounced homology to a hyaluronidase from the venom of the African Puff Adder, *Bitis arietans*, were identified in the venom of *B. flaviceps*. The low abundance (2.2%) of *B. flaviceps* hyaluronidase is consistent with observations in other venoms that this component is common but not abundant.

Nerve growth factors (NGFs) are relatively small proteins (up to 26 kDa) that induce growth and proliferation of certain neurons [45]. The NGFs encountered in snake venoms (vNGF) share significant sequence homology to their mammalian counterparts, but relatively little is known about their function and contribution to the envenomation process [46]. We detected three peptides related to vNGFs with homology to those from *B. fasciatus* and *B. multicinctus*, corroborating studies on the venoms of *B. candidus* [21], *B. multicinctus*, and *B. fasciatus* [26], in which vNGFs were also found in very low amounts.

The enzyme LAAO causes oxidative deamination of (primarily hydrophobic) L-amino acids and ultimately leads to the release of ammonia, α-keto acids and, importantly, hydrogen peroxide [47]. This compound (H_2_O_2_) is associated with various detrimental biological effects, including apoptosis induction and platelet aggregation [48]. It has also been speculated that the anti-bacterial effects of LAAOs, also likely dependent on peroxide production, may be related to maintenance and stabilization of the venom in the venom gland [43], but this seems unlikely, as several other stabilization mechanisms are known to be present [49].

### 2.2. Minor Venom Components

In the present study, there were also many toxin families present in very low abundance (up to 1.5%), some of which were detected by only a single peptide (Supplemental Table S2). The functions of some of the cysteine-rich secretory protein (CRISP or helveprin) family are related to the blockage of different ion channels, specifically, the inhibition of cyclic nucleotide gated ion channels and calcium channels, which could lead to the disruption of homeostasis in the prey [50]. While the predicted CRISP protein from the corresponding transcriptome study was not detected [25], we found peptides in the venom related to CRISP proteins from different snake families, including elapids, viperids, and colubrids, which might indicate an enhanced diversification of this toxin in *B. flaviceps* (Table 1 and Table 2). In addition, peptides related to a phospholipase B (PLB) from M. *fulvius* [38] were also found (Table 1 and Table 2). PLBs share PLA_1_ and PLA_2_ enzymatic activities and have been reported in venoms at the protein level in only a few species, including *Sistrurus catenatus edwardsii* [51], *B. candidus* [21], and *B. fasciatus* [26]. SVMPs are common components in viperid and colubrid venoms, in strong contrast to elapid venoms, where they often in much lower abundance [52]. SVMPs can be subdivided, based on in their domain structure, into different classes that are related to diverse biological activities [53]; P-III SVMPs were detected in the venom of *B. flaviceps* and accounted for 1.2% of the total spectral count (Figure 3). While most of the identified sequences were related to SVMPs of congeneric *B. multicinctus* and the elapid *Notechis scutatus*, we also identified peptides matching to SVMPs of viperid (e.g., *Bothrops asper*) and colubrid snakes, indicating an extensive diversification of this toxin that might point to accelerated evolution of this particular venom protein family in *B. flaviceps* [54]. Also, in this context, the sequence similarity search of the tandem mass spectra against a venom database proved to be especially useful, and most of the identified peptides belonged to SVMPs (Supplemental Table S2). Recent investigations of the closely related venoms *B. candidus* and *B. fasciatus* found SVMPs to account for approximately 5% of the total venom, while in the venom of *B. multicinctus* [26], the amount was rather low (0.8%). However, in these other *Bungarus* venoms, the SVMPs were much less diversified than in the *B. flaviceps* venom. In viperids, abundant SVMPs induce local and systemic hemorrhagic effects following envenomation, and we speculate that minor effects might occur following *B. flaviceps* envenomation.

Serine proteases are abundant components of many viperid venoms, and as a result of a detailed proteome analysis, they have recently been described in the venoms of *B. fasciatus* and *B. candidus* [21]. However, the first evidence of this protein family in the genus *Bungarus* stems from a study by Zhang and co-workers that identified a factor X activator in the venom of *B. fasciatus* as a serine protease [55]. In the present study, three different proteins similar to serine proteases from the pitvipers *Crotalus adamanteus*, *Sistrurus catenatus edwardsii*, and *Deinagkistrodon acutus* were detected (Table 1 and Table 2). 

Natriuretic peptides play an important role in pressure-volume homeostasis, affecting blood pressure and renal filtration, and they have been isolated from several snake venoms. For example, the venom of the Green Mamba, *Dendroaspis angusticeps,* contains a natriuretic peptide (NP) known as *Dendroaspis* natriuretic peptide (DNP), which causes vasodilation and lowers blood pressure in victims [56]. A recent proteome study of the venoms of *B. fasciatus* and *B. candidus* revealed for the first time the presence of a natriuretic peptide (NP) at the protein level in the genus *Bungarus* [21], and the authors reasoned that it might be responsible for hyponatremia observed in victims of bites from *B. candidus* [21,57]. A full-length mRNA sequence of an NP was found in the transcriptome of *B. flaviceps* [25], and a protein called KNP was characterized [58]. We were able to confirm the translation of this protein by the detection of a single semi-tryptic peptide corresponding to the predicted sequence (Table 1). NPs isolated from snake venoms, especially DNP, have greater stability and increased potency compared with mammalian NPs and are considered potential drug lead candidates [59].

Cobra venom factor (CVF) is a glycoprotein found in different cobra venoms that activates the complement system in prey [60,61]. Complement activation by CVF eventually leads to the exhaustive consumption of many compounds of the innate immune system and the weakening of the bite victim. In this study, we confirmed the presence of a peptide identical to a predicted CVF sequence from *B. fasciatus*.

### 2.3. Quantification of Venom Components

In the present study, we have quantified the snake venom protein families based on spectral count of the corresponding tandem mass spectra followed by normalization in order to take into consideration the different length of the proteins. While this protocol is sound for those proteins also identified at the transcriptome level, as their exact amino acid sequence is known, it might cause a bias for proteins detected only by sequence identity or homology from other snake venoms, because the length of these proteins in *B. flaviceps* venom is not known and can only be estimated. For example, several different SVMPs have been identified in the venom proteome, but there is no sequence information on SVMPs from the corresponding transcriptome analysis of the *B. flaviceps* venom. However, it is reasonable to assume that they belong to the PIII class (approx. 600 amino acids) of SVMPs because in most elapid venoms characterized to date, this type of SVMP is the most abundant one [62]; additionally, we detected low metalloproteinase activity in assays of the venom (data not shown).

It is also interesting to compare the method of venom protein quantification used in the present study to other quantitative snake venom investigations. Quantitative analysis of the venoms of *B. candidus* and *B. fasciatus*, for example, was based on the number of the corresponding proteins of the different protein venom families identified [21]. To estimate the abundance of each protein family, the investigators expressed these numbers (number of proteins per family) in percentage compared to the total number of identified proteins. Using yet another method, Pla and coworkers estimated the relative protein abundances by separating the crude venom with reversed-phase HPLC [63]. Individual protein abundances were calculated by dividing the single peak area by the total area of the chromatogram [63]; in cases were more than one protein was present in a chromatographic fraction, their corresponding percentage was estimated by densitometry of Coomassie-stained SDS gels. These few examples illustrate how differently snake venom proteins are quantified by different workers, and it should be stressed that there is currently no consensus in the scientific community of how best to estimate protein venom abundances. This variety of the quantification methods makes it challenging to compare different snake venom protein analyses, and it indicates that official guidelines for the experimental protocols need to be delineated in order for results from different laboratories to generate comparable results. This would lead to more robust and more readily comparable datasets, which in turn will have a profound impact on investigations into the molecular mechanisms of snake venom evolution. We would like to stress that the present study is only the view of the authors to quantify protein families and we do not claim it represents the “best” solution for this task in general.

## 3. Conclusions

We have described one of the most exhaustive characterizations of a venom proteome of an elapid snake to date, revealing a venom complexity formerly unknown to the genus *Bungarus* [21,25]. Since the currently available venom databases are largely incomplete, the use of *de novo* sequencing as a tool to characterize protein families and isoforms has proven to be particularly useful in the present study, as evidenced by the detection of 58 additional peptides. Several protein families (3FTxs, serine protease inhibitors, acetylcholinesterases, and PLA_2_s) represent the most abundant toxin classes in the venom of *B. flaviceps*, but we also detected numerous less abundant snake venom proteins, including representatives of PLB, PDE, CRISP, vNGF, hyaluronidase, natriuretic peptide, nucleotidase, VEGF-F, acetylcholinesterase, SVSP, and vespryn venom protein families. In addition, we found protein families not yet reported in krait venoms (snaclecs, and complement depleting factors), indicating that *Bungarus* venoms are more complex and diverse than previously reported. However, our results represent the venom composition of only three individual specimens from a limited part of the species’ range, and further studies are needed to determine possible geographic, gender or age-related sources of variation in the venom composition of *B. flaviceps*. The results of this study have important implications for the most effective approaches to use for generating near-complete venom proteomes, and the approaches detailed here may shed light on divergence of snake venom gland genomes and how this has affected the molecular evolution of snake venoms.

## 4. Materials and Methods

### 4.1. Venom Extraction and Ethics Statement

Three specimens of *Bungarus flaviceps* were imported from southwest Sumatra (Bushmaster Reptile; Boulder, CO, USA) and maintained at the University of Northern Colorado Animal Resource Facility in accordance with UNC-IACUC protocol #1302D-SM-S-16 (approval date: 30 March 2016). Venom was extracted manually and then centrifuged at 10,000 rpm for 5 min, frozen at −80 °C, lyophilized, and stored at −20 °C until use; venom used in this study represents the combined extractions of these three individuals: (1) male, 1405 mm snout-vent length, 240 mm tail length, total length = 1645 mm; (2) female, 940 mm snout-vent length, 168 mm tail length, total length = 1108 mm; (3) female, 1060 mm snout-vent length, 150 mm tail length, total length = 1210 mm. Snakes were extracted four times each and the average yield was 24.7 L. Range = 5–75 L. No animals were sacrificed during this study.

### 4.2. Tryptic Digestion of Crude Venom

Lyophilized venom (100 µg) was dissolved in 40 L of 0.4 M ammonium bicarbonate and 8 M urea. After adding 10 L of 50 mM dithiothreitol (DTT), the solution was incubated for 3 h at 37 °C. Additionally, 10 µL of iodoacetamide (150 mM) was added at room temperature and the reaction was allowed to proceed for 15 min. The reaction was quenched with 50 mM of DTT (6 µL) for 15 min. The last two steps were performed in the dark. For digestion, the sample solution was diluted to 1 M urea (by adding 254 µL of water) and incubated with 10 µL (2 µg in 50 mM acetic acid) of trypsin (Promega, Madison, WI, USA) at 37 °C overnight. Quenching of the reaction occurred by adding trifluoroacetic acid (40 µL), and desalting of the sample was performed on Poros R2 microcolumns. As a final step, the peptides were dried in a vacuum centrifuge and brought up in 1% formic acid solution (approx. 50 µL).

### 4.3. Chromatography and Mass Spectrometry

#### 4.3.1. One-Dimensional Sodium Dodecyl Sulfate Polyacrylamide Gel Electrophoresis (SDS-PAGE)

Crude venom was reduced with 2.5% β-mercaptoethanol in Laemmli sample buffer (Bio-Rad, Hercules, CA, USA.) by incubation at 95 °C for 10 min. Samples (20 µg and 50 µg amounts) were run on a 12% Bis-Tris acrylamide gel, stained with 0.1% Coomassie Brilliant Blue R-250 overnight, and destained in 10% acetic acid: 40% methanol: 50% ddH_2_0. A Novex Mark 12 unstained mass standard (Life Technologies, Grand Island, NY, USA) was also run for band mass estimation. Protein bands were excised and submitted to the Protein and Proteomics Centre in the Department of Biological Sciences, National University of Singapore, for LC-MS/MS analysis.

#### 4.3.2. Liquid Chromatography-Tandem Mass Spectrometry (LC-MS/MS) Analysis of Excised Gel Bands

Bands were reduced with 10 mM DTT (dithiothreitol) and alkylated with 55 mM IAA (iodoacetamide), then digested with trypsin (13 ng/µL) overnight in 25 mM ammonium bicarbonate, 10% ACN (acetonitrile). Samples were desalted using a Sep-Pak tC18 μ Elution Plate (Waters, Milford, MA, USA), and reconstituted with 20 L of diluent (97.5% H_2_O, 2% ACN, 0.05% formic acid). Peptide separation was carried out on an Eksigent nanoLC Ultra and ChiPLC-nanoflexLC-MS (Eksigent, Dublin, CA, USA) in Trap Elute configuration. A total of 5 L of the sample was loaded onto a 200 μm × 0.5 mm trap column and eluted on an analytical 75 μm × 150 mm column. Trap and analytical columns were made of ChromXP C18-CL, 3 μm (Eksigent, Dublin, CA, USA). Peptides were separated by a gradient formed by 2% ACN, 0.1% FA (mobile phase A) and 98% ACN, 0.1% FA (mobile phase B); 5 to 7% of mobile phase B in 0.1 min, 7 to 30% of mobile phase B in 10 min, 30 to 60% of mobile phase B in 4 min, 60 to 90% of mobile phase B in 1 min, 90 to 90% of mobile phase B in 5 min, 90 to 5% of mobile phase B in 1 min and 5% of mobile phase B for 10 min, at a flow rate of 300 nL/min. The MS analysis was performed on a TripleTOF 5600 system (AB SCIEX, Redwood City, CA, USA) in Information Dependent Mode. MS spectra were acquired across the mass range of 400–1250 *m*/*z* in high resolution mode (>30,000) using 250 ms accumulation time per spectrum. A maximum of 10 precursors per cycle were chosen for fragmentation from each MS spectrum with 100 ms minimum accumulation time for each precursor and dynamic exclusion for 8 s. Tandem mass spectra were recorded in high sensitivity mode (resolution > 15,000) with rolling collision energy on adjustment. Survey-IDA experiment with charge states 2 to 4, which exceeds 125 cps, was selected. Peptide identification was achieved with ProteinPilot 5.0 software Revision 4769 (AB SCIEX, Redwood City, CA, USA) using the Paragon database search algorithm (5.0.0.0.4767) for peptide identification and the integrated false discovery rate (FDR) analysis function. The data were searched against a database consisting of SerpentesDB database (total 345,092 entries). The search parameters are as follows: Sample Type: Identification; Cys Alkylation: Iodoacetamide; Digestion: Trypsin; Special Factors: None; Species: None. The processing was specified as follows: ID Focus: Biological Modifications; Search Effort: Thorough; Detected Protein Threshold: 0.05 (10.0%); and Competitor Error Margin (ProtScore): 2.00.

#### 4.3.3. Electrospray Mass Spectrometry of Digested Venom

Approximately 300 ng of trypsin-digested venom was applied to a nano-chromatography system (Ultimate 3000 system, Thermo Scientific Dionex, Sunnyvale, CA, USA) connected to an electrospray mass spectrometer (Q Exactive™ Plus Hybrid Quadrupole-Orbitrap™, Thermo Scientific, Waltham, MA, USA). The chromatography consisted of an initial desalting and concentration step of the sample using a capillary “trap” column (2 cm × 100 m i.d.) packed with 5 m, 200 Å Magic C18 AQ matrix (Michrom Bioresources, Auburn, CA, USA). The fractionation of the peptides was performed on a 30 cm analytical column (75 m i.d., packed with 1.9 m ReproSil-Pur 120 C18-AQ; Dr. Maisch) including a laser-pulled tip (~5 m). Using standard mobile phases (solvent A: 0.1% formic acid in water; solvent B: 0.1% formic acid in acetonitrile), we applied a shallow gradient of 2–40% of B over 160 min for maximum peptide resolution. To elute possible hydrophobic peptides, the gradient was increased to 80% B over 4 min at the end of the run. The capillary temperature was set to 250 °C and a spray voltage of 1.9 kV with no sheath or auxiliary gas flow was applied. During the acquisition of the mass spectrometric data, up to 12 of the most intense precursor ions were selected for fragmentation using higher energy collisional dissociation (HCD). Normalized collision energy of 30% was applied, and singly-charged peptides or peptides with unresolved charge states were excluded. The acquisition parameters for full MS were as follows: resolution 70,000 (FWHM, full width at half-maximum, at *m*/*z* 200), automatic gain control pre-scan (AGC) target 1 × 10^6^, maximum ion injection time (IT) of 100 ms, scan range 300–1500 *m*/*z*, and profile spectrum data type. Data-dependent (dd) MS/MS acquisition parameters were as follows: resolution 17,500, AGC target 5 × 10^4^, maximum injection time 50 ms, isolation window 2.0 *m*/*z* (with 0.5 *m*/*z* offset). Dynamic exclusion time was set to 60 s. For external calibration, a mixture of caffeine, peptide MRFA (Met–Arg–Phe–Ala) and Ultramark 1621 was used following the manufacturer’s instructions.

#### 4.3.4. MALDI Mass Spectrometry of Digested Venom

The tryptic digest was separated on a C18 reversed phase column (Agilent Zorbax 300SB-C18 1.0 × 150 mm × 3.5 m) by running a linear gradient from 0% to 50% acetonitrile over 120 min applying a flow rate of 40 L/min (solvent A 0.1% TFA, solvent B contains 80% ACN with 0.1% TFA) on an Eksigent system (Dionex, Vernon Hills, IL, USA). During the chromatographic run, approximately 200 fractions were manually collected directly onto the MALDI plate and 0.3 µL of a saturated matrix solution (10 mg/mL α-cyano-4-hydroxycinnamic acid (Aldrich, Milwaukee, WI, USA) in 50% acetonitrile with 0.1% TFA was added. Finally, the solution was dried at room temperature (dried-droplet method). Raw data for protein identification were obtained on an AB Sciex 5800 (AB Sciex, Foster City, CA, USA). Up to 15 of the most intense ion signals with a signal-to-noise ratio above 2 were selected as precursors for MS/MS acquisition (excluding common trypsin autolysis and keratin peaks). External calibration in MS mode was performed using a mixture of six singly charged peptides: des-Arg1-Bradykinin (*m*/*z* = 904.468), angiotensin I (*m*/*z* = 1296.685), Glu1-fibrinopeptideB (*m*/*z* = 1570.677), ACTH (1–17 clip; *m*/*z* = 2093.087), ACTH (18–39 clip; *m*/*z* = 2465.199), and ACTH (7–38 clip; *m*/*z* = 3657.929). MS/MS spectra were externally calibrated using known fragment ion masses observed in the tandem mass spectrum of Glu1-fibrinopeptideB.

### 4.4. Data Analysis of Digested Venom

Database searches of the mass spectra acquired by MALDI or electrospray MS were searched against a database compiled of venom sequences deposited on NCBI (71798 entries, 8 June 2015) using Mascot version 2.1 (Matrix Science Inc., Boston, MA, USA). The following search parameters were applied: no restrictions on species of origin or protein molecular weight, two tryptic missed cleavages allowed, variable modifications of cysteine (carbamidomethylation) and methionine (oxidation), and pyro-glutamate formation at N-terminal glutamine of peptides. The enzyme conditions were set to “semi-tryptic” including the option to search for non-tryptic cleavage sites either at the N-terminus or C-terminus of the corresponding peptide. The database search of electrospray data (Orbitrap Q Exactive™, ThermoFisher Scientific, Waltham, MA, USA) applying a *p*-value of 0.05% showed a false discovery rate (FDR) of 1.82%, while the database search of spectra acquired with MALDI mass spectrometry revealed a FDR of 0.49% (*p*-value 0.05%).

### 4.5. De Novo Sequencing and Similarity-Driven Analysis of Digested Venom

In addition to automated database searches, all tandem mass spectra were sequenced by de novo analysis using the program PEAKS version 6 (Bioinformatics Solutions Inc., Waterloo, KW, Canada). The following search parameters were applied: cysteine modification (carbamidomethylation of cysteine), deamidation of asparagine (N), oxidation of methionine, and pyroglutamate modification at N-terminal glutamate; 20 ppm precursor mass tolerance and 0.1 Da fragment mass tolerance. High quality de novo sequences (Peaks ALC score higher than 50 and sequences with six or more amino acid residues) were submitted to the algorithm PepExplorer [64] to identify possible snake venom peptides not detected by automated database searches (against a snake venom database including 71,798 entries). The search included the PAM (point accepted mutation) 30MS substitution matrix to score the alignments. Initially, peptides with at least 65% of sequence identity compared with a sequence from the database were accepted. Finally, tandem mass spectra of sequences that satisfied the above mentioned criteria were manually confirmed

### 4.6. Protein Quantitation

Relative protein abundances were estimated by normalized spectral-counts of the corresponding MS/MS spectra, referred to as the normalized spectral abundance factor (NSAF) [65].

## Figures and Tables

**Table 1:** Snake venom protein families of *B. flaviceps* identified by automated database search. SVMP—snake venom metalloproteinase; SVSP—snake venom serine proteinase.

Protein Family	Protein	Accession No.	Species	Number of Peptides Matched
**3FTx**	Non-conventional three finger toxin isoform 1	294961050	*Bungarus flaviceps*	6
**3FTx**	Non-conventional three finger toxin isoform 6	294961060	*Bungarus flaviceps*	5
**3FTx**	Short-chain three finger toxin isoform 4	294961042	*Bungarus flaviceps*	4
**3FTx**	Short-chain three finger toxin isoform 7	294961048	*Bungarus flaviceps*	9
**3FTx**	Short-chain three finger toxin isoform 6	294961046	*Bungarus flaviceps*	7
**3FTx**	Short-chain three finger toxin isoform 1	294961036	*Bungarus flaviceps*	2
**3FTx**	κ-bungarotoxin	809178	*Bungarus multicinctus*	1
**3FTx**	Short-chain three finger toxin isoform 3	294961040	*Bungarus flaviceps*	1
**3FTx**	κ-flavitoxin	128938	*Bungarus flaviceps*	9
**3FTx**	Muscarinic toxin-like protein	294961066	*Bungarus flaviceps*	9
**Serine protease inhibitor**	β-bungarotoxin B chain precursor	31745053	*Bungarus flaviceps*	7
**Serine protease inhibitor**	Kunitz-type serine proteinase inhibitor isoform 5	294961076	*Bungarus flaviceps*	8
**Serine protease inhibitor**	Kunitz-type serine proteinase inhibitor isoform 1	294961068	*Bungarus flaviceps*	4
**Acetylcholinesterase**	Acetylcholinesterase	1389604	*Bungarus flaviceps*	20
**Acetylcholinesterase**	Acetylcholinesterase DEN	476538388	*Denisoni adevisi*	4
**PLA_2_**	β-bungarotoxin A_2_ chain precursor	31745049	*Bungarus flaviceps*	16
**PLA_2_**	β-bungarotoxin A_1_ chain precursor	31745051	*Bungarus flaviceps*	3
**PLA_2_**	Phospholipase A_2_II precursor	31745057	*Bungarus flaviceps*	21
**PLA_2_**	Phospholipase A_2_	263083	*Bungarus fasciatus*	8
**PLA_2_**	Phospholipase A2 precursor	31745055	*Bungarus flaviceps*	23
**PLA_2_**	Phospholipase A_2_ isoform 3	294961092	*Bungarus flaviceps*	5
**PLA_2_**	Phospholipase A_2_Kbf-III	110559306	*Bungarus fasciatus*	4
**PLA_2_**	Phospholipase A_2_ isozyme 1	24638470	*Laticauda semifasciata*	1
**PLA_2_**	Phospholipase A_2_	29422777	*Bungarus candidus*	2
**PLA_2_**	Phospholipase A_2_	5924345	*Austrelaps superbus*	3
**PLA_2_**	Phospholipase A_2_	152032644	*Bungarus fasciatus*	3
**PLA_2_**	Phospholipase A_2_ precursor	156257593	*Bungarus fasciatus*	1
**PLA_2_**	Phospholipase A_2_	48425218	*Bungarus caeruleus*	1
**PLA_2_**	Phospholipase A_2_	129428	*Laticauda colubrina*	1
**VEGF**	Hypothetical protein L345_04144	565318860	*Ophiophagus hannah*	4
**Nucleotidase**	Ecto-5′-nucleotidase 1	537444870	*Micrurus fulvius*	11
**Snaclec**	Snaclec factor IX/factor X-binding protein B chain	398488	*Bothrops jararaca*	1
**Snaclec**	C-type lectin-like protein 1	13876735	*Bungarus fasciatus*	3
**Vespryn**	Ohanin precursor	70907886	*Ophiophagus hannah*	2
**Vespryn**	Vespryn22	336042222	*Drysdalia coronoides*	1
**Phosphodiesterase**	Phosphodiesterase 1	537444868	*Micrurus fulvius*	20
**Phosphodiesterase**	Phosphodiesterase 1	338855302	*Crotalus adamanteus*	1
**Hyaluronidase**	Hyaluronidase	113203681	*Bitis arietans*	4
**VNGF**	Venom nerve growth factor precursor	266299	*Bungarus multicinctus*	4
**LAAO**	L-amino-acid oxidase	126035653	*Bungarus fasciatus*	5
**LAAO**	L-amino acid oxidase	126035649	*Bungarus multicinctus*	2
**LAAO**	L-amino-acid oxidase	426205815	*Crotalus durissus cumanensis*	2
**PLB**	Phospholipase B	537444729	*Micrurus fulvius*	3
**CRISP**	Cysteine-rich seceretory protein	190195343	*Bungarus candidus*	3
**CRISP**	Opharin precursor	225547744	*Ophiophagus hannah*	2
**SVMP**	Scutatease-1 (PIII)	145982766	*Notechis scutatus*	8
**SVMP**	Metalloproteinase (PIII)	126035640	*Bungarus multicinctus*	6
**SVMP**	Metalloproteinase MTP9 (PIII)	336042214	*Drysdalia coronoides*	4
**SVMP**	Metalloproteinase (PIII)	126035635	*Bungarus fasciatus*	4
**SVMP**	P-III	633276509	*Micropechis ikaheka*	4
**SVMP**	MTP4 (PIII)	537463069	*Micrurus fulvius*	3
**SVMP**	Atragin precursor(PIII)	224482347	*Naja atra*	3
**SVMP**	Metalloproteinase isoform 3 (PIII)	109254964	*Sistrurus catenatus edwardsi*	2
**SVMP**	SVMP-Hop-14, partial (PIII)	476539284	*Hoplocephalus bungaroides*	2
**SVMP**	SVMP-Hop-46, partial(PIII)	476539268	*Hoplocephalus bungaroides*	2
**SVMP**	SVMP 1	537444726	*Micrurus fulvius*	2
**SVMP**	Metalloproteinase (PII)	82466485	*Bothrops asper*	1
**SVMP**	Fur-1, partial (PI)	476538467	*Furinaor nata*	1
**SVMP**	jararhagin (PIII)	62468	*Bothrops jararaca*	1
**SVMP**	Metalloproteinase (PIII)	241995585	*Philodrya solfersii*	1
**SVMP**	Leucurolysin-B (PIII)	223635807	*Bothrops leucurus*	1
**SVMP**	Ech-32 (PIII)	476538400	*Echiopsis curta*	1
**SVMP**	Cobrin precursor(PIII)	6006966	*Naja naja*	1
**SVMP**	Metalloproteinase (PII)	297594122	*Echis pyramidum leakeyi*	1
**SVMP**	CohPH-3 (PII)	522802426	*Crotalus oreganus helleri*	1
**SVSP**	Serine proteinase isoform 2	109254940	*Sistrurus catenatus edwardsi*	2
**SVSP**	SVSP 11	387014258	*Crotalus adamanteus*	1
**Natriuretic peptide**	Natriuretic peptide	294961100	*Bungarus flaviceps*	1
**Complement-depleting factor**	Complement-depleting factor	126035660	*Bungarus fasciatus*	1

**Table 2:** Snake venom protein families of *B. flaviceps* identified by de novo sequencing of tandem mass spectra followed by sequence-similarity analysis. The corresponding sequences are indicated.

Protein Family	Protein	Accession No.	Species	Database Sequence	Alignment Score	De Novo Sequence	ALC (%)	*m*/*z*	ppm to De Novo Derived Sequence
**3FTx**	Muscarinic toxin-like protein	117606606	*Ophiophagus hannah*	TFTCPELTPN	64	CGTYTCPELTPDRR	52	575.9354	13.5
**PLA_2_**	Phospholipase A_2_	9453880	*Laticauda semifasciata*	VHDDCYGEAEK	87	VHDDCYGEAEK	58	563.5938	−1.8
				LVQFTYLIQCANKGSRASYHYADYGCY	84	RLAQFACLLQCADEEVGVDLHYADYGCYNCK	46	906.911	5.8
**Nucleotidase**	Snake venom 5′-nucleotidase	752779244	*Boiga irregularis*	VVQFMNSLR	69	EAAAVVQFMNSLR	51	479.2537	7.1
				HGQGTGELLQVSGIKVVYDLS	99	HPQGTGELLQVSLGKVVYDLSKGAPVNK	52	587.7292	4.2
**Snaclec**	C-type lectin 2	537444936	*Micrurus fulvius*	DHHCPSDWYSFDKFCYKFI	105	DNHCNADWSSFDKFCYQFLR	52	653.2799	2
**Vespryn**	Vespryn 23	336042224	*Drysdalia coronoides*	HFFEVK	49	HFFEVK	60	403.7129	−1.1
				IVVFLDYSEGK	68	LVVFLDYKEGK	60	437.5828	−1.2
				IVVFLDY	51	LVVFLDYK	71	498.791	−1.7
**Phosphodiesterase**	Phosphodiesterase	675402318	*Protobothrops elegans*	FYTLYIEEPDTTGHK	106	QFLPVFFTLYLEEPDTTGHK	57	592.0533	7
				RPDFYTLYIEEPDTTGHK	130	RPDFFTLYLEEPDTTGHK	59	542.2665	−2.6
**VNGF**	Venom nerve growth factor	335892638	*Bungarus fasciatus*	YFFETK	51	AGYFFETK	51	481.7344	−0.2
**LAAO**	L-amino acid oxidase	126035653	*Bungarus fasciatus*	SYVTADYVIVCAT	76	LTSYVTADYLLVSATMGR	51	981.0001	−6.1
				TADYVIVCATSR	80	TLSAAHTADYLLVCATSR	52	650.6723	14.1
**LAAO**	L-amino acid oxidase	401021343	*Lachesis muta*	LSAAYVLAEAGHQVTVLEASER	105	LSAAYVLSEKHQVTVLQASER	52	583.3127	−2.5
**LAAO**	L-amino acid oxidase	537444909	*Micrurus fulvius*	SAAYVLAEAGHKVTLLE	103	QLLVVQGMQDSAAYVLSEAGHKVTVLESNNK	50	669.7629	20.9
				AGMAGLSAAYVLAEAGHK	116	VKHHSVPPRVAGMAGLSAAYVLSEAGHK	52	574.7169	10.5
**LAAO**	L-amino acid oxidase	3426324	*Crotalus adamanteus*	RVVIVGAGMAGLSAAYVL	104	RLALVGAGMAGLSAAYVLNPSSPGVLSQLLWSR	53	671.7708	−3.1
**LAAO**	L-amino acid oxidase 1a	537444909	*Micrurus fulvius*	GMAGLSAAYVLAEAGHK	110	RTPVVKGMAGLSAAYVLSEAGHK	48	590.3197	−1.2
**Phospholipase B**	Phospholipase B	537444729	*Micrurus fulvius*	KGYWPSYNIPFHK	94	KPYWPSYNLPFHK	56	559.6232	−1.9
**CRISP**	CRISP precursor	158262802	*Austrelaps superbus*	VDKHNALR	58	VAAVDKHNALR	45	398.5624	−2
				YLYVCQYCPAGNIRGSIATPYK	84	YLYVCQYCFRAFGAPYNNKHGSLATPYK	43	847.8989	−8.6
**CRISP**	Cysteine-rich secretory protein A	698375481	*Opheodrys aestivus*	WNSNAAQNAK	76	EMWNSNAAQNAK	44	682.3055	−1.5
				PAGNIVGSIATPYK	95	MENTQAAVCSDPAGNLVGSLATPYK	44	846.4114	7.4
				PAGNIVGSIATPYK	95	LYYCMNEGQMAPAGNLVGSLATPYK	44	897.7457	−14.4
				ASCFCH	50	HCASCFCHGGK	48	632.2381	−10.1
**SVSP**	Venom thrombin-like enzyme	118500915	*Deinagkistrodon acutus*	DECDINEHR	72	VVNDECDLNEHR	51	500.5556	−1.3
**SVMP**	Metalloproteinase 13b	699236656	*Hypsiglena* sp. *JMG-2014*	DPDNGMVEPGTK	82	LNMMSSSPNACADPDEGMVEPGTK	50	846.6896	−2.9
**SVMP**	Metalloproteinase 4a	752782853	*Boiga irregularis*	YLVVDNR	53	LYLVVDNR	72	496.282	−0.4
				AYEMVNILNVIFR	81	AYEMVNLLNTMFR	61	801.3932	−1.5
				YLVVDNR	53	TNTARCLTLYLKLKYQPPAYLVVDNR	51	622.9447	4
				SPDYGMVEPGTK	64	DYPGHHACALEDDQLLMCSEDYGMVEPASK	51	850.1182	11.6
**SVMP**	Metalloproteinase	172046653	*Naja mossambica*	TSMVAITMAHQMGHNLGMNDDR	122	NTDMVAGTMAHQMGHNLGLNDHR	52	841.0485	7.2
				AHQMGHNLGMNDDR	81	EFVRQAFANALYPPWWMPKYMVPQKCKAHEMGHNLGLNDHR	50	986.0939	12.5
**SVMP**	Metalloproteinase (type III) 2a	752782867	*Boiga irregularis*	TEGMVEPGTK	75	QTEGMVEPGTK	60	580.2688	0.3
				TESFVASTMAHELGHNLGINHDR	136	TNPFVGSTMAHEMGHNLGLNHDR	60	634.5504	7.2
**SVMP**	Atrase B, partial	289655973	*Naja atra*	FGEWRETVLLPR	92	AFGEWRETVLLPR	63	525.2878	0.2
**SVMP**	Metalloproteinase 11a	699236668	*Hypsiglena* sp. *JMG-2014*	QPCQNNQGYCYNGK	94	GPGLQPCGNNQGYCYDGK	44	964.4064	−0.3
				KYIEFYIVVDH	78	SMTKPCLMRVCDLVKYLQFYLVVDHVKSYVLCYR	48	823.6322	11.2

## Supplementary Materials

The following are available online at http://www.mdpi.com/2072-6651/10/9/373/s1, Table S1: Proteins identified in different Coomassie-stained bands of a 1D gel of the crude venom of *B. flaviceps*, Table S2: Proteins identified in the venom of *B. flaviceps* including detailed information on peptide sequences, *m*/*z* values, and ppm values.
