# Supplementary material for: Proteomic Deep Mining the Venom of the Red-Headed Krait, Bungarus flaviceps
Source: Toxins (Basel). 2018 Sep 13;10(9):373. doi: 10.3390/toxins10090373 (PMC6162843; doi:10.3390/toxins10090373)
Supplement: Supplementary file 1 [file toxins-10-00373-s001.zip › toxins-322780-supp-proofreading/Bungarus_flaviceps_supplemental_Table_2.pdf]

Supplemental Table 2.

Protein family	Protein	Accession no.	Species	Peptide	m/z	∆ppm
**3FTx**	Non-conventional three finger toxin isoform 1	294961050	*Bungarus flaviceps*	E.SWSTAR.G	354.1763	-2.52
				K.ESWSTAR.G	418.6975	-2.4
				K.VCSGEEIYCFK.E	696.3013	-1.25
				K.ICQFNTCRPGELK.V	811.8916	-1.13
				K.VCSGEEIYCFKESWSTAR.G	736.9929	-2.19
				K.ICQFNTCRPGELKVCSGEEIYCFK.E	749.8442	4.74
**3FTx**	Non-conventional three finger toxin isoform 6	294961060	*Bungarus flaviceps*	R.FYEGK.R	322.1574	-1.63
				T.LTCLICPEK.Y	567.2878	-0.81
				T.LTCLICPEKYCQK.V	571.6105	0.02
				R.GCAATCPEAKPHEIVECCSTDK.C	*2520.07*	*2.27*
				R.GCAATCPEAKPHEIVECCSTDKCNK.-	*2922.26*	*9.44*
**3FTx**	Short-chain three finger toxin isoform 4	294961042	*Bungarus flaviceps*	K.MYFSDHR.G	319.1407	-1.52
				R.GCVATCPTNNR.Y	625.2732	-1.7
				R.GCVATCPTNNRYDR.V	562.2427	-1.85
				T.RICYNQQSTTPPTTENCEPGKNVCYK.M	787.1072	-2.12
**3FTx**	Short-chain three finger toxin isoform 7	294961048	*Bungarus flaviceps*	R.GCIAACPK.P	438.706	-2.11
				K.NEMIQCCSK.D	585.2402	-1.45
				R.GCIAACPKPK.K	551.28	-1.41
				K.KNEMIQCCSK.D	649.2877	-1.29
				K.RGCIAACPKPK.K	315.1688	-1.77
				K.TCPSGQNVCFK.K	649.2855	-2.12
				K.KNEMIQCCSKDK.C	514.5628	-1.13
				K.TCPSGQNVCFKK.W	*1425.66*	*-0.03*
				R.DNESSKTCPSGQNVCFK.K	653.2844	0.34
3FTx	Short-chain three finger toxin isoform 6	294961046	*Bungarus flaviceps*	K.NSWIYR.G	419.7129	-2.35
				R.GCSLTCPDIK.S	575.765	-0.43
				K.SNGKYIYCCTR.D	474.8755	-1.55
				K.LCYNHQSTNPK.T	454.5462	-1.84
				T.KLCYNHQSTNPK.T	*1489.72*	*-0.14*
				R.GCSLTCPDIKSNGK.Y	513.2361	-2.99
				K.TTELCGHSMYFCYK.N	*1796.74*	*1.85*
**3FTx**	Short-chain three finger toxin isoform 1	294961036	*Bungarus flaviceps*	K.YIYCCTR.D	518.2225	-0.95
				T.NVCYTHESANPK.T	473.8815	-1.47
**3FTx**	κ-bungarotoxin	809178	*Bungarus multicinctus*	R.SLLCCTTDNCNH.-	748.2842	0.11
**3FTx**	Short-chain three finger toxin isoform 3	294961040	*Bungarus flaviceps*	K.SSWIYR.G	406.2072	-3.15
**3FTx**	κ-flavitoxin	128938	*Bungarus flaviceps*	K.AFCDR.W	334.644	-1.99
				K.GQDICFTK.A	484.7288	-0.31
				K.SLLCCTTDNCNH.-	748.2842	0.11
				K.GQDICFTKAFCDR.W	539.9088	-1.94
				R.TCLISPSSTSQTCPK.G	833.8908	-1.96
				-.RTCLISPSSTSQTCPK.G	608.2971	-0.98
				R.GPVIEQGCAATCPEFTSR.Y	*1979.9*	*0.51*
				-.RTCLISPSSTSQTCPKGQDICFTK.A	693.8319	-2.09
				K.AFCDRWCSSRGPVIEQGCAATCPEFTSR.Y	827.3643	5.1

**3FTx**	Muscarinic toxin-like protein	294961066	*Bungarus flaviceps*	K.KYPVMLK.R	439.7615	-1.13
				K.ILICCPTR.N	516.77	-0.23
				K.KYPVMLKR.G	345.5437	-1.31
				R.GCVTTCTGPK.G	540.7433	-1.71
				K.SYWVNEFGNK.Q	*1243.57*	*0.48*
				R.CCPEGQDSCYK.S	702.2548	-0.06
				M.CVRPYPFDSEDR.C	514.2328	-0.59
				R.MCNMCVRPYPFDSEDR.C	698.2838	1.12
				T.RMCNMCVRPYPFDSEDR.C	562.9902	1.51
**Serine protease inhibitor**	β-bungarotoxin B chain precursor	31745053	*Bungarus flaviceps*	R.FSYGGCK.G	409.6784	-0.75
				R.HPDCDKPPNK.K	403.1884	-1.35
				R.CTGHIPAFYYN.P	671.7983	0.6
				K.TPQLCMCHCHE.-	736.7795	-1.43
				K.RHPDCDKPPNK.K	455.2215	-2.56
				R.CTGHIPAFYYNPQR.K	575.2709	-1.67
				R.CTGHIPAFYYNPQRK.T	*1851.9*	*0.66*
**Serine protease inhibitor**	Kunitz-type serine protease inhibitor isoform 5	294961076	*Bungarus flaviceps*	A.FYYHPASNK.C	563.7713	3.31
				K.FAFYYHPASNK.C	*1344.63*	*0.13*
				G.GCGGNDNNFK.T	541.7185	-2.08
				R.KFAFYYHPASNK.C	736.869	-0.9
				K.YCNLPPEPGPCHGR.K	551.913	-1.05
				K.YCNLPPEPGPCHGRK.F	594.6114	-0.78
				K.EFVYGGCGGNDNNFK.T	839.8401	-4.29
				K.CKEFVYGGCGGNDNNFK.T	*1965.81*	*-3.83*
**Serine protease inhibito**	Kunitz-type serine protease inhibitor isoform 1	294961068	*Bungarus flaviceps*	R.VYYYIPAAR.K	558.2967	-1.93
				K.RVYYYIPAAR.K	*1271.69*	*1.04*
				R.KCEQFIYGGCK.G	*1389.63*	*-0.12*
				L.GVPEYCNLPPDPGPCDAYKR.V	*2305.04*	*0.0*
**Acetylcholinesterase**	Acetylcholinesterase	1389604	*Bungarus flaviceps*	R.ADFLEGVR.M	453.7366	-2.24
				V.YAYLFDHR.A	362.1779	-1.69
				K.VSTQTGSVR.G	467.7505	-1.66
				K.VYAYLFDHR.A	395.2009	-1.03
				K.ETQVLLGVVK.D	543.3312	-1.19
				K.LLNATVDPPR.A	548.311	-0.92
				E.LKVSTQTGSVR.G	392.5623	-1.74
				K.LLNATVDPPRA.D	583.8295	-0.96
				K.LLNATVDPPRAD.-	641.3424	-1.71
				R.AQICAFWNHFLPK.L	544.9366	-2.42
				R.AQICAFWNHFLPKL.L	582.3047	0.19
				K.DEGSYFLIYGLPGFSK.D	598.2967	-2.07
				K.NPQELIDEEWSVLPYK.S	980.4896	1.04
				K.QLGCHFNNDSELVSCLR.S	677.9699	-2.93
				R.SKNPQELIDEEWSVLPYK.S	725.7041	0.44
				R.FLRPEPVKPWQHVLDATSYK.P	804.4313	-3.22
				R.AILQSGGPNAPWATVTPAESR.G	708.3672	-0.45
				K.DEGSYFLIYGLPGFSKDNESLISR.A	903.1122	-1.03
				R.MRFLRPEPVKPWQHVLDATSYK.P	679.3591	-3.34
				R.VGAFGFLGLPGSPEAPGNMGLLDQR.L	834.4276	7.58

**Acetylcholinesterase**	Acetylcholinesterase DEN−1	476538388	*Denisoniadevisi*	N.NKVYAYLFDHR.A	475.9136	-0.68
				R.AILQSGGPNAPWATVTPAESR.R	*2123.1*	*7.55*
				S.AVTIFGESAGAASVGMHLLSTQSR.A	797.4089	-0.51
				R.GLSLPVLDGHVSAFLGIPFAEPPVGR.M	882.4869	0.41
**PLA_2**	β-bungarotoxin A_2 chain precursor	31745049	*Bungarus flaviceps*	P.IDALDR.C	351.6921	-1.51
				K.VVSYSSK.C	385.2072	-2.39
				S.LNLLQFK.D	438.2704	-1.52
				R.CTIPCER.T	468.2068	-1.18
				R.FVCDCDR.T	486.1886	-1.03
				L.LQFKDMIR.C	525.7927	1.64
				L.NLLQFKDMIR.C	639.3586	5.15
				S.LNLLQFKDMIR.C	464.2672	-0.42
				R.TLFCYDAPGSCAR.F	759.3281	-1.53
				R.TAALCFGDSEYIGR.H	780.3607	-1.09
				K.RTLFCYDAPGSCAR.F	558.5884	-1.01
				R.CCYVHDNCYGDAEKR.N	649.5897	-1.65
				A.SNIPPQSLNLLQFKDMIR.C	706.3754	7.98
				R.TWGEYADYGCYCGAGGSGR.P	696.2728	1.02
				R.FVCDCDRTAALCFGDSEYIGR.H	838.0253	-3.09
				R.TWGEYADYGCYCGAGGSGRPIDALDR.C	*2867.24*	*8.79*
**PLA_2**	β-bungarotoxin A_1 chain precursor	31745051	*Bungarus flaviceps*	K.GGSGRPVDALDR.C	400.5422	-0.62
				R.CCYVHDNCYGEAQK.R	601.9003	-1.06
				R.CCYVHDNCYGEAQKR.N	*1959.79*	*-1.15*
**PLA_2**	Phospholipase A_2II precursor	31745057	*Bungarus flaviceps*	K.CSPYYK.T	409.1806	-1.25
				D.YGCYCGK.G	454.1750	-1.08
				A.DYGCYCGK.G	511.6888	-0.34
				K.TYKYDCSEGK.L	625.7708	-1.14
				K.LTCNDAPGSCER.S	690.2856	-2.86
				R.VAAICFAGAPYN.D	627.3018	-1.55
				K.GGGGTPVDELDR.C	586.7801	-1.13
				R.VAAICFAGAPYNDK.N	748.8632	-0.70
				R.CCQTHDNCYGEAEK.L	591.2197	-1.27
				R.ATWHYADYGCYCGK.G	876.3512	0.59
				R.VAAICFAGAPYNDKN.F	805.8862	1.20
				L.NLIQFSSLIQCANGGSR.A	933.4729	11.3
				R.CCQTHDNCYGEAEKLTK.C	705.2940	-2.97
				R.VAAICFAGAPYNDKNF.M	879.4216	2.52
				R.VAAICFAGAPYNDKNFM.I	944.9362	-3.64
				K.TYKYDCSEGKLTCNDAPGSCER.S	871.0300	-3.91
				R.SVCDCDRVAAICFAGAPYNDK.N	797.0167	-0.65
				R.SVCDCDRVAAICFAGAPYNDKN.F	835.0339	2.95
				K.GGGGTPVDELDRCCQTHDNCYGEAEK.L	*2925.22*	*14.0*
				R.ATWHYADYGCYCGKGGGGTPVDELDR.C	727.0647	2.23
				L.NLIQFSSLIQCANGGSRATWHYADYGCYCGKGGGGTPVDELDR.C	951.4310	2.52

**PLA$_2$**	Phospholipase A_2	263083	*Bungarus fasciatus*	D.VDLETR.C	366.6975	-1.39
				R.TAALCFAGAP.Y	489.7386	-1.39
				K.SMVQCTSTRPWL.D	733.8500	11.0
				K.SMVQCTSTRPWLD.Y	790.8622	-1.58
				A.PYNQNNFDVDLETR.C	863.8793	-2.43
				R.TAALCFAGAPYNQNNF.D	879.8990	0.16
				R.TAALCFAGAPYNQNNFDVDLETR.C	*2587.18*	*-2.04*
				R.TAALCFAGAPYNQNNFDVDLETRCQ.-	959.0962	-1.18
**PLA$_2$**	Phospholipase A2 precursor	31745055	*Bungarus flaviceps*	N.FFISFK.E	394.7224	4.17
				D.NFFISFK.E	451.7415	-1.58
				R.SVCDCDR.V	456.1702	-1.61
				Y.AHYGCYCGK.G	558.2226	-1.6
				H.YAHYGCYCGK.G	639.7544	-1.2
				K.YDCSEGKLTCK.D	680.7963	-1.3
				K.LTCKDAPGSCER.S	*1392.6207*	*0.61*
				K.GGGGTAVDELDR.C	573.7725	-0.71
				K.TYKYDCSEGKLTCK.D	584.9348	-1.85
				R.PTWHYAHYGCYCGK.G	600.5853	-0.25
				N.RPTWHYAHYGCYCGK.G	489.7163	0.3
				R.CCQTHDNCYGEAEKLPK.C	528.2242	-1.67
				K.YDCSEGKLTCKDAPGSCER.S	744.9813	-1.08
				H.GNRPTWHYAHYGCYCGK.G	709.9676	-1.41
				R.VAANCFAGAPYNNDNFFISFK.E	789.7009	0.76
				R.SVCDCDRVAANCFAGAPYNN.D	*2260.9194*	*1.13*
				R.VAANCFAGAPYNNDNFFISFKEN.C	870.7296	0.89
				M.IQCANHGNRPTWHYAHYGCYCGK.G	713.3166	8.89
				R.VAANCFAGAPYNNDNFFISFKENCQ.-	966.7565	-2.12
				K.GGGGTAVDELDRCCQTHDNCYGEAEKLPK.C	*3237.41*	*3.93*
				R.SVCDCDRVAANCFAGAPYNNDNFFISFKENCQ.-	*3790.7*	*32.1*
				L.NLYQFNNMIQCANHGNRPTWHYAHYGCYCGK.G	*3875.64*	*-1.03*
				Q.PLNLYQFNNMIQCANHGNRPTWHYAHYGCYCGK.G	682.1191	-17.98
**PLA$_2$**	Phospholipase A_2 isoform 3	294961092	*Bungarus flaviceps*	R.FVCDCDRVAAN.C	663.7806	-2.01
				A.GAPYNDKNFMINFK.T	553.6046	-0.14
				R.CCQTHDNCYDEAEKLTK.C	543.7244	-1.46
				R.VAANCFAGAPYNDKNFMINFK.T	803.7106	7.12
				L.NLIQFSSLIQCANGGSRPTWHYADYGCYCGK.G	*3623.6895*	*17.7*
**PLA$_2$**	Phospholipase A_2Kbf-III	110559306	*Bungarus fasciatus*	K.NMIQCAGTR.S	525.7441	-0.97
				S.YDCSEGKLTCK.A	*1360.588*	*0.62*
				K.NMIQCAGTRS.W	569.2602	-0.73
				-.NLFQFKNMIQCAGTRS.W	639.3154	8.49
**PLA$_2$**	Phospholipase A_2 isozyme 1	24638470	*Laticauda semifasciata*	L.NLVQFSNLIQCVNK.G	839.4205	-17.33
**PLA$_2$**	Phospholipase A_2	29422777	*Bungarus candidus*	R.TAALCFAEAPYK.R	671.3289	-0.21
				R.TAALCFAEAPYKR.R	749.3789	-0.94
**PLA$_2$**	Phospholipase A_2	5924345	*Austrelaps superbus*	F.VCDCDVEAAK.C	583.744	-0.75
				R.FVCDCDVEAAK.C	657.2776	-1.64
				R.FVCDCDVEAAKCFAR.A	616.6002	-0.67

**PLA$_2$**	Phospholipase A$_2$	152032644	*Bungarus fasciatus*	K.CSSLLNVPYVK.Q	640.3388	-0.89
				R.TAALCFAEVPYK.R	685.3411	-5.15
				K.CSSLLNVPYVKQYSYTCSEGNLTCSADNDECAAFICNCDR.T	949.3816	-15.15
**PLA$_2$**	Phospholipase A$_2$ precursor	156257593	*Bungarus fasciatus*	L.NLFQFKNMIECAGTR.S	914.9537	9.19
**PLA$_2$**	Phospholipase A$_2$	48425218	*Bungarus caeruleus*	-.NLYQLMNMIQCAN.T	815.3601	3.62
**PLA$_2$**	Phospholipase A$_2$	129428	*Laticauda colubrina*	R.ATYYYMDYGCYCGK.G	915.8485	4.89
**VEGF**	Hypothetical protein L345_04144	565318860	*Ophiophagus hannah*	K.QLELNER.T	451.2393	-2.7
				K.HLYKQDPLTCK.C	468.2431	0.01
				P.DEVITFMTVFER.S	743.8662	0.33
				K.HFQSQHIHPMSFQQHSK.C	526.7531	-1.25
**Nucleotidase**	Ecto-5'-nucleotidase 1	537444870	*Micrurus fulvius*	K.SIQEDPAVK.A	493.7606	-1.34
				K.ASGNPILLNK.S	513.8	-1.45
				K.FPILSANIRPK.G	419.2554	-0.42
				K.LTILHTNDVHAR.V	348.1948	-1.63
				Q.VPVVQAYAFGK.Y	589.8316	-0.77
				R.VVSLNVLCTECR.V	725.3618	-1.72
				K.IIALGHSGFKEDCR.I	401.4565	-1.06
				K.NVKFPILSANIRPK.G	399.9945	-1.44
				K.SIQEDPAVKAEVSR.M	510.2699	-0.97
				K.VLLPSFLAAGGDGYYMLK.G	639.0057	-0.37
				R.YDAMALGNHEFDNGLNGLLDPLLK.N	878.4308	12.9
**Snaclec**	Snaclec factor IX/factor X-binding protein B chain	398488	*Bothrops jararaca*	-.DCPSDWSPYEGHCYR.V	643.5813	-1.72
**Snaclec**	C-type lectin-like protein 1	13876735	*Bungarus fasciatus*	K.YIWEWTDR.S	584.7767	3.33
				C.YTCPIDWLPK.N	646.8193	-2.29
				K.FKPGCHLASLHSNADAVEFSEYISDYLTGQGHVWIGLR.D	856.0187	2.09
**Vespryn**	Ohanin precursor	70907886	*Ophiophagus hannah*	R.EWAVGLAGK.S	465.7552	-1.32
				K.ADVTFDSNTAFESLVVSPDKK.T	757.3772	-0.73
**Vespryn**	Vespryn22	336042222	*Drysdalia coronoides*	*K.TVENVGVPQVVPDNPER.F*	*1848.94*	*-1.37*
**Phosphodiesterase**	Phosphodiesterase 1	537444868	*Micrurus fulvius*	R.QWLAVR.N	386.726	-2.33
				K.SLQMADR.T	410.7023	-0.93
				K.TFLPIFVNSVN.-	625.8428	0.34
				R.TLGMLMEGLK.Q	546.7946	2.58
				R.IDKVNLMVDR.Q	401.5567	-0.43
				K.SPNNLWVEER.M	622.7979	-1.72
				K.DFYTFDSEAIVK.N	717.844	1.37
				R.LKTFLPIFVNSVN.-	746.9336	12.8
				K.AATYFWPGSEVK.I	678.3335	-2.43
				R.LWNYFHSTLLPK.Y	759.907	-2.41
				R.TLGMLMEGLKQR.N	459.584	-0.98
				R.MANVLCSCSEDCLTK.K	894.3743	-1.02
				K.NVPKDFYTFDSEAIVK.N	625.3174	8.52
				K.SLQMADRTLGMLMEGLK.Q	637.3231	0.13
				K.YISAYSQDILMPLWNSYTISK.S	831.7507	0.26
				K.SLQMADRTLGMLMEGLKQR.N	545.5359	8.53
				R.NLHNCVNLILLADHGMEAISCNR.L	666.8252	-0.49
				R.VRDVELLTGLDFYSVLKQPLSETLR.L	723.6427	-13.39
				R.NGLNVISGPIFDYNYDGHFDSYDTIK.Q	989.1311	8.8
				K.VLSFILPHRPDNSESCADKSPNNLWVEER.M	682.934	-0.78

**Phosphodiesterase**	Phosphodiesterase 1	338855302	*Crotalus adamanteus*	R.NPAWWGGQPIWHTATYQGLK.A	771.0519	0.17
**Hyaluronidase**	Hyaluronidase	113203681	*Bitis arietans*	R.NDQLLWLWR.D	622.3322	-0.85
				R.EDYALPVFVYAR.P	721.8697	0.47
				K.HSDSNAFLHLFPESFR.I	476.7323	-0.69
				K.TFHGLGVIDWENWRPQWDR.N	603.7956	-1.12
**VNGF**	Venom nerve growth factor precursor	266299	*Bungarus multicinctus*	R.IDTACVCVISR.K	647.3177	-0.68
				R.FIRIDTACVCVISR.K	570.6304	-3.52
				R.HWNSYCTTTDTFVK.A	587.26	-5.11
				R.HWNSYCTTTDTFVKA.L	610.941	-1.65
**LAAO**	L-amino-acid oxidase	126035653	*Bungarus fasciatus*	R.SALEECFR.E	506.2316	-0.52
				R.EADYEEFLEIAR.N	*1484.69*	*3.09*
				K.TSADIVINDLSLIHQLPK.N	989.0513	-3.09
				R.SALEECFREADYEEFLEIAR.N	826.3818	0.76
				R.VNTYRDEKEGWYVNMGPMR.L	782.3628	-2.61
**LAAO**	L-amino acid oxidase	126035649	*Bungarus multicinctus*	*R.IYFAGEYTAR.V*	*1190.58*	*-1.45*
				K.IQHDAEKVR.V	365.8679	-1.19
**LAAO**	L-amino-acid oxidase	426205815	*Crotalus durissus cumanensis*	Q.LNEFFQENENAWYFIK.N	1046.4948	0.57
				L.QLNEFFQENENAWYFIK.N	740.6972	16.9
**PLB**	Phospholipase B	537444729	*Micrurus fulvius*	K.QVVPESLFAWER.V	730.8803	-0.01
				A.DLHYATVYWLEAEK.S	579.9537	-1.02
				R.NAGYIIAQLDGLYMGNLEWAKR.Q	838.7606	13.5
**CRISP**	Cysteine-rich seceretory protein	190195343	*Bungarus candidus*	R.CQTEWIK.S	482.731	-0.94
				K.QIVDKHNALR.R	*1193.68*	*6.2*
				I.FSCGENLFMSSQPYAWSK.V	1069.9666	-2.43
**CRISP**	Opharin precursor	225547744	*Ophiophagus hannah*	Q.MEWNSNAAQNAK.R	682.3055	-1.52
				K.FSCGENLFMSSQPYAWSR.V	1083.9828	9.76
**SVMP**	Scutatease-1 (PIII)	145982766	*Notechis scutatus*	R.ETVLLPR.K	414.2522	-1.76
				R.DRPQCIL.N	451.2308	-1.80
				L.TNTPEQDR.Y	480.7221	-1.34
				K.WRETVLLPR.K	*1169.6766*	*-2.0*
				R.NDNAQLLTGI.E	529.7835	11.3
				R.DRPQCILNKPLSTD.I	828.9215	-1.05
				K.LQHEAQCDSEECCEQCK.F	553.4692	15.5
				I.ISFEPLSEFSSCSVQEHQR.Y	756.3429	-11.28
**SVMP**	Metalloproteinase (PIII)	126035640	*Bungarus multicinctus*	K.KLLPR.K	313.7206	-1.53
				R.NDNAQLLTR.I	522.7742	-2.07
				R.SACCNAATCK.L	571.7224	-1.13
				K.RNDNAQLLTR.I	601.3170	-1.41
				R.KRNDNAQLLTR.I	333.1858	-1.57
				S.MVASTMAHEMGHNLGINHDR.A	556.0071	0.37
**SVMP**	Metalloproteinase MTP9 (PIII)	336042214	*Drysdalia coronoides*	*Q.SVAVIQDHSKR.T*	*1239.68*	*-0.08*
				Q.SAECPTDSFQR.N	*1297.54*	*-3.12*
				R.NGHPCQNNQGYCYNGK.C	637.5919	-2.34
				T.ISNQPLSEFSSCSVQEHQR.Y	*2234.03*	*8.41*

**SVMP**	Metalloproteinase (PIII)	126035635	*Bungarus fasciatus*	H.EMGHNLGINHDR.A	348.9158	-1.75
				N.YFVEVGEECDCGSPR.D	*1803.73*	*-1.32*
				R.NGLPCQNNQGYCYNGK.C	943.8995	0.45
				S.MVASTMAHEMGHNLGINHDR.A	556.0071	0.37
**SVMP**	P-III	633276509	*Micropechis ikaheka*	D.LPEICTGR.S	473.2435	-2.58
				K.DDCDLPEICTGR.S	725.7994	-1.54
				L.TNTPEQDRYLQVK.K	*1592.8*	*4.11*
				R.AAKDDCDLPEICTGR.S	*1720.76*	*-0.23*
**SVMP**	MTP4 (PIII)	537463069	*Micrurus fulvius*	K.NDCDLPELCTGR.S	725.7994	-1.53
				P.LYEFSSCSVQQHQR.Y	590.6011	-1.29
				S.DEPLYEFSSCSVQQHQR.Y	*2110.92*	*2.58*
**SVMP**	Atragin precursor(PIII)	224482347	*Naja atra*	K.CGDGMVCSNR.Q	578.2201	-0.94
				R.TKPAYQFSSCSVR.E	765.8715	-0.84
				R.NGLPCQNNQGYCYNGK.C	944.8829	-0.14
**SVMP**	Metalloproteinase isoform 3 (PIII)	109254964	*Sistrurus catenatus edwardsi*	*S.CTGQSADCPTDDLHR.N*	*1715.69*	*8.95*
				N.ELLEVGEECDCGSPNTCR.D	*2124.88*	*9.52*
**SVMP**	SVMP-Hop-14, partial (PIII)	476539284	*Hoplocephalus bungaroides*	G.IDFNGTTVGR.A	540.7695	-0.34
				K.KSVAVIQDHSKR.T	456.5958	-1.92
**SVMP**	SVMP-Hop-46, partial(PIII)	476539268	*Hoplocephalus bungaroides*	L.AYVGTLCSPK.F	548.2783	-0.97
				F.SVAVIQNYSR.D	569.2978	-1.35
**SVMP**	SVMP 1	537444726	*Micrurus fulvius*	*R.AAKDDCDLPELCTGR.S*	*1720.76*	*-0.23*
				K.GLFSEDYTETHYAPDGR.I	653.2891	-1.76
**SVMP**	Metalloproteinase (PII)	82466485	*Bothrops asper*	*K.YNSNLNTIR.T*	*1094.5745*	*14.3*
**SVMP**	Fur-1, partial (PI)	476538467	*Furina ornata*	S.SVAVIQDYSR.R	569.2978	-1.37
**SVMP**	jararhagin (PIII)	62468	*Bothrops jararaca*	R.MYELANIVNEIFR.Y	814.4091	-2.44
**SVMP**	Metalloproteinase (PIII)	241995585	*Philodryas olfersii*	D.PDNGMVEPGTK.C	572.7673	-2.55
**SVMP**	Leucurolysin-B (PIII)	223635807	*Bothrops leucurus*	Q.SAQCPTDDFKR.N	663.2929	-0.37
**SVMP**	Ech-32 (PIII)	476538400	*Echiopsis curta*	*L.TYTPEQNRYLQIKK.Y*	*1782.9335*	*-2.77*
**SVMP**	Cobrin precursor(PIII)	6006966	*Naja naja*	K.LQHEAQCDSEECCEK.C	641.5815	-0.75
**SVMP**	Metalloproteinase (PII)	297594122	*Echis pyramidum leakeyi*	K.SVGIVQDHSKEHLLVAATMAHEMGHNLGMKHDGD.Q	614.2999	7.22
**SVMP**	CohPH-3 (PII)	522802426	*Crotalus oreganus helleri*	L.AYVGSMCHPKRSTGIIQDYSPINLVVAVIMAHEMGHNLGINHDR.G	701.0615	-7.11
**SVSP**	Serine proteinase isoform 2	109254940	*Sistrurus catenatus edwardsi*	*R.AAYPEYGLPATSR.T*	*1395.7*	*11.1*
				L.SLPSSPPSVGSVCR.I	*1429.72*	*9.45*
**SVSP**	SVSP 11	387014258	*Crotalus adamanteus*	L.VVGGDECNINEHR.S	500.5556	-1.33
**Natriuretic peptide**	Natriuretic peptide	294961100	*Bungarus flaviceps*	I.SHTSDMGCR.H	350.8073	-1.6
**Complement-depleting factor**	Complement-depleting factor	126035660	*Bungarus fasciatus*	R.THNIEGTSYALLALLK.M	581.9935	1.11
